# The polymorphic variant rs1800734 influences methylation acquisition and allele-specific TFAP4 binding in the MLH1 promoter leading to differential mRNA expression

**DOI:** 10.1038/s41598-019-49952-x

**Published:** 2019-09-17

**Authors:** Rachael Thomas, Davide Trapani, Lily Goodyer-Sait, Marketa Tomkova, Ceres Fernandez-Rozadilla, Nora Sahnane, Connor Woolley, Hayley Davis, Laura Chegwidden, Skirmantas Kriaucionis, Timothy Maughan, Simon Leedham, Claire Palles, Daniela Furlan, Ian Tomlinson, Annabelle Lewis

**Affiliations:** 1 Cancer Gene Regulation Group, Wellcome Trust Centre for Human Genetics, University of Oxford, Roosevelt Drive, Oxford, OX3 7BN UK; 2 Anatomic Pathology Unit, Department of Medicine and Surgery and Research Center of Hereditary and Familial Tumors, University of Insubria, Varese, 21100 Italy; 3 Institute of Structural and Molecular Biology, Department of, Biological Sciences, Birkbeck, London, UK; 4 Ludwig Institute for Cancer Research Ltd, University of Oxford, Nuffield Department of Clinical Medicine, Old Road Campus Research Building, Roosevelt Drive, Oxford, OX3 7DQ UK; 5 Fundación Pública Galega de Medicina Xenómica, Grupo de Medicina Xenómica, IDIS, Santiago de Compostela, Spain; 6 Cancer Genetics and Evolution Laboratory, Institute of Cancer and Genomic Sciences, College of Medical and Dental Sciences, University of Birmingham, Birmingham, UK; 7 Intestinal Stem Cell Biology Group, Wellcome Trust Centre for Human Genetics, Oxford University, Roosevelt Drive, Oxford, OX3 7BN UK; 8 Gastrointestinal Cancer Genetics Laboratory, Institute of Cancer and Genomic Sciences, College of Medical and Dental Sciences, University of Birmingham, Birmingham, UK; 9 Oxford Institute of Radiation Oncology, University of Oxford, Old Road Campus Research Building, Roosevelt Drive, Oxford, OX3 7DQ UK; 10 Division of Biosciences, Department of Life Sciences, Brunel University London, Old Road Campus Research Building, Roosevelt Drive, Uxbridge, UB8 3PN UK

**Keywords:** Cancer genetics, Cancer epigenetics, Cancer genomics, Functional genomics, Chromatin

## Abstract

Expression of the mismatch repair gene MutL homolog 1 (MLH1) is silenced in a clinically important subgroup of sporadic colorectal cancers. These cancers exhibit hypermutability with microsatellite instability (MSI) and differ from microsatellite-stable (MSS) colorectal cancers in both prognosis and response to therapies. Loss of *MLH1* is usually due to epigenetic silencing with associated promoter methylation; coding somatic mutations rarely occur. Here we use the presence of a colorectal cancer (CRC) risk variant (rs1800734) within the *MLH1* promoter to investigate the poorly understood mechanisms of *MLH1* promoter methylation and loss of expression. We confirm the association of rs1800734 with MSI+ but not MSS cancer risk in our own data and by meta-analysis. Using sensitive allele-specific detection methods, we demonstrate that MLH1 is the target gene for rs1800734 mediated cancer risk. In normal colon tissue, small allele-specific differences exist only in MLH1 promoter methylation, but not gene expression. In contrast, allele-specific differences in both *MLH1* methylation and expression are present in MSI+ cancers. We show that *MLH1* transcriptional repression is dependent on DNA methylation and can be reversed by a methylation inhibitor. The rs1800734 allele influences the rate of methylation loss and amount of re-expression. The transcription factor TFAP4 binds to the rs1800734 region but with much weaker binding to the risk than the protective allele. TFAP4 binding is absent on both alleles when promoter methylation is present. Thus we propose that TFAP4 binding shields the protective rs1800734 allele of the MLH1 promoter from BRAF induced DNA methylation more effectively than the risk allele.

## Introduction

Colorectal cancer (CRC) is a major cause of morbidity and mortality, with a lifetime risk of ~6% in the UK. About 15% of sporadic CRCs are deficient in DNA mismatch repair (MMR), a process that normally acts to correct spontaneous DNA replication errors. MMR-deficient cancers exhibit a high rate of mutation genome-wide, but this is most evident at short repeat sequences, causing these cancers to be termed “microsatellite-unstable” (MSI+). The majority of sporadic MSI+ cancers have reduced protein and mRNA expression of the MMR gene, MutL homolog 1 (*MLH1*). This loss of *MLH1* is rarely caused by mutations. Instead, epigenetic silencing occurs, with high levels of DNA methylation present in the *MLH1* promoter. Understanding how MSI+ CRCs develop is clinically important. Stage II/III MSI+ CRCs have a relatively favourable prognosis, whereas stage IV MSI+ tumours have a poor prognosis. MSI+ CRCs respond poorly to commonly used chemotherapy, such as 5-fluorouracil but are targetable by immune checkpoint inhibitors due to their high number of neoantigens^1,2^.

The importance of *MLH1* in CRC and its propensity for hypermethylation have been known for some time^3^, and there is a substantial body of literature about the use of *MLH1* methylation and/or MSI as a biomarker in the classification of CRC. However, the biological mechanisms underlying the methylation have not been investigated in detail until recently. Fang *et al*.^4^ have demonstrated in cancer cell lines that BRAF oncogenic mutations mediate the CpG island methylation phenotype (CIMP) resulting in hypermethylation at *MLH1* and other CIMP marker genes, via the transcriptional repressor MAFG^4^.

Some further understanding of the acquisition of *MLH1* promoter methylation has come from the study of a single nucleotide polymorphism (SNP), rs1800734, that lies in the 5′ untranslated region of *MLH1*. An association between rs1800734 and CRC risk has been shown in multiple candidate studies^5–8^. However, this strong association is limited to MSI+ cancers, and is weak or absent in un-stratified data sets. Several groups have investigated possible mechanisms by which this SNP may confer increased CRC risk, although the results are not straightforward. There is an association between the rs1800734 risk (A) allele and (i) DNA methylation at the *MLH1* promoter in cancers^9,10^, and (ii) CpG island shore methylation in normal tissue^11,12^. There is also evidence that binding of the transcription factor TFAP4 (AP-4) is modified by rs1800734^13,14^ *in vitro* and *in vivo*. However, Liu *et al*.^13^ detected no difference in *MLH1* allele-specific expression as a result of TFAP4 allelic bias. Instead, they showed an effect on the expression of the gene encoding the protein kinase DCLK3 and long-range chromatin interactions between rs1800734 and the DCLK3 promoter.

Here we confirm the association of rs1800734 with CRC risk in our own MSI+ data set, confirm its absence in MSS cancers and perform a meta-analysis with other publically available MSI+ datasets. We then describe a comprehensive study which confirms *MLH1* as the target gene, and investigate the relationship between the rs1800734 allele, *MLH1* promoter methylation and, importantly *MLH1* mRNA expression in normal tissue and the pathway to colon cancer. To add to this correlative data, we demonstrate the causal role of methylation by dynamically altering *MLH1* promoter methylation levels, and showing that methylation loss and gain is modified by the rs1800734 allele with downstream effects on mRNA expression and TFAP4 binding.

## Results

### rs1800734 is strongly associated with MSI+ positive CRC risk but not MSS cancers

The *MLH1* promoter SNP rs1800734 has been assessed as a candidate for CRC susceptibility in a number of MSI+ colorectal cancer data sets. We confirmed these results in our own MSI+ data set from the VICTOR and QUASAR2 CRC clinical trials (n = 170, Supplementary Table 1 OR = 1.95, 95%CI 1.50–2.55, p = 8.04 × 10^−7^). A meta-analysis of these data and 5 other data sets showed a highly significant association with CRC risk (OR = 1.50; 95% CI 1.34, 1.66; Pmeta < 10^−10^; 6640 cases and 8645 controls^5–8^. If an allelic model is used AG heterozygotes are at ~1.3-fold increased risk of MSI+ CRC compared with GG homozygotes, and AA homozygotes at ~2.6-fold raised risk. These are the largest effect sizes of any known common CRC predisposition SNP. Notably, MSI negative CRC cases show no significant association (OR = 1.03, p = 0.133) strongly suggesting that the SNP plays a mechanistic role in the silencing of *MLH1* during MSI+ cancer development.

### The risk allele of rs1800734 is associated with allele-specific CpG island shore methylation in normal bowel tissue, but no consistent bias in MLH1 mRNA expression

**Figure 1:**
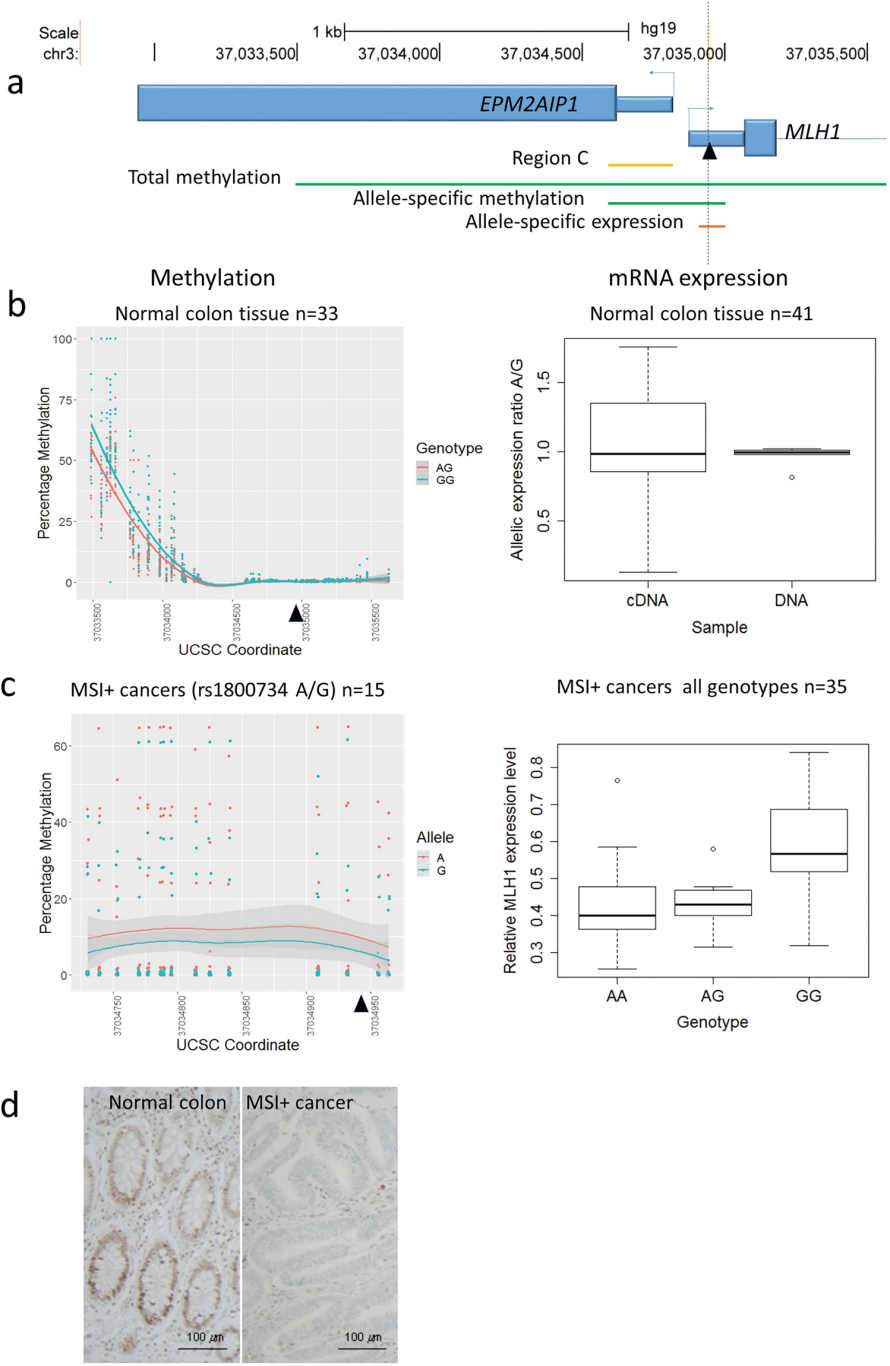
Allele specific methylation and expression of rs1800734 in the 5′ UTR of *MLH1* is seen in MSI+ cancers and SSAs but not in normal bowel. (**a**) Map of the promoter regions of *MLH1* showing the chromosomal location of rs1800734 (chr3:37034946 (hg19), black triangle and dotted line, hg19), exon 1 of *MLH1*, the gene *EPM2AIP*, Deng region C (yellow line) and regions assessed for overall methylation (long green), allele specific methylation (short green) and allele specific expression (orange). (**b**) Normal bowel: (left panel) scatter plot showing total methylation levels across all CpGs in the region with samples grouped by genotype. Loess curves for each genotype are shown with standard error shaded in grey; (right panel) boxplot showing *MLH1* allelic mRNA expression ratio (A/G) in heterozygous samples comparing cDNA with genomic DNA. (**c**) Cancer: (left panel) scatter plot showing allele specific methylation levels across CpGs close to rs1800734 with samples grouped by allele. Loess curves for each allele are shown with standard error shaded in grey; (right panel) boxplot showing total *MLH1* mRNA levels in all samples grouped by genotype. (**d**) Loss of MLH1 protein in MSI CRC (ID Number 15-I-25344 in Supplementary Table 3): Immunohistochemistry was carried out using Ventana monoclonal antibodies anti-MLH1 (clone M1) on VENTANA BenchMark ULTRA platform. A case was considered immuno-negative (right panel; 200X, scale bar = 100 µm) when all of the tumour cell nuclei or a defined cluster of tumor cells failed to react with the specific antibody, with an intact nuclear staining of mixed non-tumor cells. Normal residual colorectal mucosa of the same sample was MLH1 immunopositive (left panel; 200X, scale bar = 100 µm).

We devised a comprehensive analysis of the relationship between rs1800734 genotype, *MLH1* promoter methylation and *MLH1* mRNA expression in normal bowel (Supplementary Table 2), MSI+ CRCs (Supplementary Table 3) and sessile serrated adenomas (SSAs, Supplementary Table 4)), the putative precursor lesion of MSI+ CRCs (Fig. 1a). Figure 1b shows the methylation across the *MLH1* CpG island and shore in normal colon tissue determined by next generation sequencing (NGS) of amplicons of bisulphite-treated DNA. As expected, there was little or no methylation close to rs1800734 or in Deng region C (used for clinical evaluation of *MLH1* promoter methylation in MSI+ cancers^15^) in any of the samples. There were, however, increasing levels of methylation towards the upstream CpG island shore and interestingly these were higher in low-risk GG homozygotes than in AG heterozygotes (n = 39, Supplementary Table 2 (labelled green), p = 0.011, ANOVA; insufficient AA samples were present for statistical analysis). Though contrary to expectations, this is in agreement with previous studies^12^. We therefore wished to investigate any possible impact of this differential methylation on mRNA expression in our normal tissue samples. Since, we expected any allele-specific effects to be relatively small in this normal tissue without significant methylation in Deng region C, we developed a sensitive technique able to detect small allelic biases in cDNA by using next generation sequencing. We used heterozygous patients to carry out allele-specific expression analysis by reverse transcriptase PCR across the 5′ UTR region containing rs1800734 followed by sequencing and allelic counting. This allowed us to measure the ratio of risk to protective allele within an individual patient sample. Figure 1b shows that the mRNA allelic ratio was variable, but did not differ significantly from 1:1 in these patients (n = 41, Supplementary Table 2 (labelled yellow) p = 0.1495 T-Test). The allelic ratio from genomic DNA (1:1) was shown to be 1:1 confirming the absence of copy number changes or PCR amplification bias. We also found no eQTL at rs1800734 in our sample set (Fernandez-Rozadilla unpublished) or in publically available GTEx intestinal data sets (GTex Data Portal), which taken together suggest that rs1800734 does not exert any effect on mRNA expression in normal colon tissue despite influencing DNA methylation at the CpG island shore.

### In MSI+ cancers the risk (A) allele of rs1800734 is associated with increased methylation and lower levels of expression

Similar analyses were carried out in tumours from MSI+ cancer patients (collected at the Department of Pathology of ASST dei Sette Laghi, University of Insubria) with loss of MLH1 protein expression (Fig. 1d, Supplementary Table 3). Methylation levels as determined by MS-MLPA were variable but still significantly higher in AA and AG patients than GG patients across the CpG island (n = 35, p = 0.0002, ANOVA, Supplementary Fig. 1). We interrogated these samples further to determine if these differences were specifically due to increased levels on the A allele in heterozygous patients, again using bisulphite-treated DNA amplified by PCR, followed by 250 bp paired-end reads and MiSeq NGS (Illumina) to allow phasing of rs1800734 allele with methylation in region C. Despite variable levels of methylation between patients, in the 12 heterozygous patients, 15 samples in total, we found methylation on the A allele was significantly greater than the G allele (Fig. 1c, n = 15 Supplementary Table 3 (rs1800734 genotype AG), p = 0.0322, ANOVA). In patients of all 3 genotypes, mRNA expression levels also varied significantly with genotype (Fig. 1c, n = 35, Supplementary Table 3, p = 0.0001, ANOVA). Expression levels were significantly correlated with methylation levels (p = 1.67 × 10^−5^, Pearson), suggesting a causal relationship.

**Figure 2:**
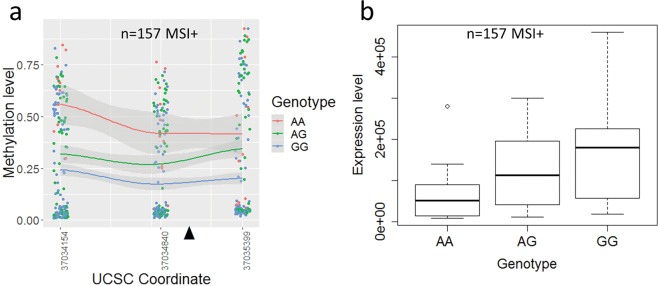
Allele specific methylation and expression of rs1800734 in the 5′ UTR of *MLH1* in MSI+ colorectal cancers from The Cancer Genome Atlas (left panel) scatter plot showing differential methylation levels across CpGs close to rs1800734 with samples grouped by genotype. Loess curves for each genotype are shown with standard error shaded in grey. rs1800734 (chr3:37034946 (hg19)), is shown by the black triangle; (right panel) boxplot showing total *MLH1* mRNA levels in all samples grouped by genotype.

We wished to validate our findings on a 2^nd^ larger dataset with a broader demographic. We therefore carried out a similar analysis on colorectal cancer with matched normal data from The Cancer Genome Atlas (TCGA COADREAD, http://cancergenome.nih.gov/). We found that methylation in region C and at rs1800734 was significantly higher in tumours with AA and AG genotypes (Supplementary Fig. 2a, n = 432, p = 0.000133 ANOVA). When stratified by MSI status the MSI+ tumours alone still showed a genotype specific significant difference in methylation levels (Fig. 2, n = 157, p = 0.00115), however MSS tumours showed no allele specific differences (Supplementary Fig. 2b, n = 275, p = 0.627). Interestingly there was also some difference in methylation in normal tissue samples taken from cancer patients (Supplementary Fig. 2c, n = 31, p = 0.0071), which differed from our findings in normal tissue taken from control patients. Analysis of mRNA expression levels on the same data sets showed that these also varied significantly with genotype in all tumour samples (Supplementary Fig. 2d, n = 432, p = 0.00153, ANOVA). When stratified by MSI status the MSI+ tumour samples alone showed a highly significant difference between the genotypes (Fig. 2, n = 157, p = 0.0006) whereas the MSS tumour samples showed no effect of genotype on expression (Supplementary Fig. 2e, n = 275, p = 0.627). Normal tissue showed no variation at all (Supplementary Fig. 2f, n = 31, p = 0.99). As in our MSI+ cancer data set, expression and methylation showed a highly significant correlation (p-value < 10^−15^, Pearson). Early lesions in the pathway to cancer could represent intermediate stages in the process of MLH1 silencing. Sessile serrated adenomas (SSAs) are known precursors of *BRAF*-mutant MSI+ CRCs^16^. SSAs that develop dysplasia progress rapidly to cancer and approximately 75% of these have methylated *MLH1* promoters and silencing^17^. Fennel *et al*.^18^ have shown that the rs1800734 risk (A) allele is associated with a dosage dependent increase in methylation in SSAs with dysplasia. The AA genotype was also associated with protein loss as measured by immunohistochemistry. We hypothesised that SSAs would show allele-specific expression at the mRNA level, so we investigated allele-specific *MLH1* mRNA expression and methylation in a small set of fresh-frozen rs1800734-heterozygous SSAs with BRAFV600E mutations (n = 5, Supplementary Table 4, Supplementary Fig. 3). Our sample set lacked power to demonstrate allele specific methylation (p = 0.120 T-test, Supplementary Fig. 3a). However the gradient of methylation across the region is clearly visible with a significant difference between region C (chr3: 37034600-37034800) and the region encompassing rs1800734 (chr3: 37034900-37035100, p < 10^−5^, T-test), possibly indicating that hypermethylation is spreading from the CpG island shore across region C towards rs1800734. The intermediate levels of methylation present in our SSAs are already sufficient to cause a difference in allelic mRNA expression levels (p = 0.039, T-Test, Supplementary Fig. 3b) Taken together with the findings of Fennel *et al*.^18^, the combined data suggest that rs1800734 genotype can influence DNA methylation and mRNA transcription at an early stage in the serrated pathway to colorectal cancer.

### Removal of DNA methylation causes de-repression of MLH1 transcription with an allele-specific bias

**Figure 3:**
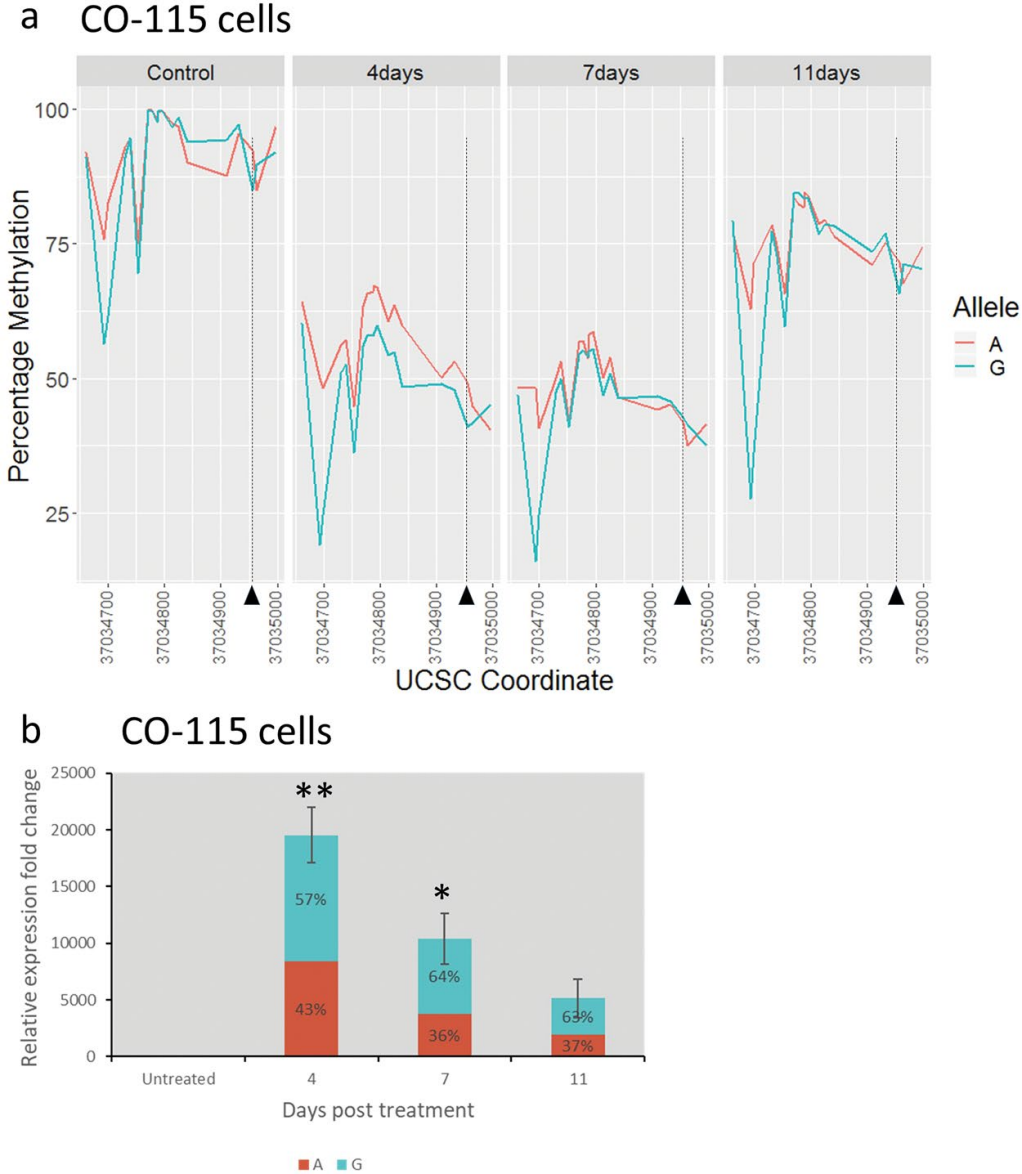
Allele specific analysis of MLH1 demethylation and derepression after AzaC treatment of CO-115 cells. (**a**) Line-graphs showing percentage methylation levels at individual CpGs in the rs1800734 region grouped by allele. Each panel shows a control or time-point post AzaC treatment. The position of rs1800734 is marked (chr3:37034946 (hg19), black triangle). (**b**) Barchart showing total *MLH1* mRNA expression and the allelic components of this expression in control cells and time-points post AzaC treatment. Error bars show the standard error of the mean of replicates. Asterisks denote significant (*p < 0.05) or highly significant (*p < 0.01) increases in expression in AzaC treated cells compared with untreated.

The MSI+ CRC cell line CO-115 (heterozygous for rs1800734 (A/G), BRAFV600E) has very high levels of methylation at all CpGs analysed in the *MLH1* promoter (Fig. 3a, mean = 91%) and expression of *MLH1* is undetectable by Q-PCR (Fig. 3b). Hypermethylation is observed on both the A and G alleles although some CpGs show lower levels on the G allele. We hypothesized that the untreated cells represented the endpoint state, after promoter methylation has been completed. We wished to take a dynamic approach to determine if DNA methylation is the primary and causal event leading to MLH1 transcriptional silencing and to observe how the rs1800734 allele affects the rate of loss and gain of methylation. 5-azacytidine and 5-aza-2′-deoxycytidine are chemical analogues of cytosine and inhibitors of DNA methylation. They cause a global loss of methylation when used to treat cell lines^19^. When treated with 5 μM 5-aza-2′-deoxycytidine (AzaC) for 48 hours, we saw a reduction in DNA methylation at all CpGs (mean methylation = 50%, p < 1 × 10^−15^ ANOVA), which gradually increased again over 11 days (Fig. 3a). Interestingly at all stages of the experiment we observed significantly higher levels of methylation on the risk (A) allele (p = 0.0137, two-way ANOVA), with the A both taking longer to lose its methylation and regaining it more quickly. The loss of *MLH1* methylation was accompanied by re-expression of *MLH1* mRNA, peaking at 4 days post-AzaC treatment (Fig. 3b; p = 0.007, ANOVA) and then starting to reduce by day 11 post-treatment. Again, there were allele-specific differences at each stage, with the risk (A) allele expressed at lower levels than the protective (G). Re-expression of *MLH1* to similar levels was also seen in a second MSI+ CRC cell line, SW48, following 5AzaC-induced loss of methylation (Supplementary Fig. 4).

### TFAP4 binding at rs1800734 is allele-specific

**Figure 4:**
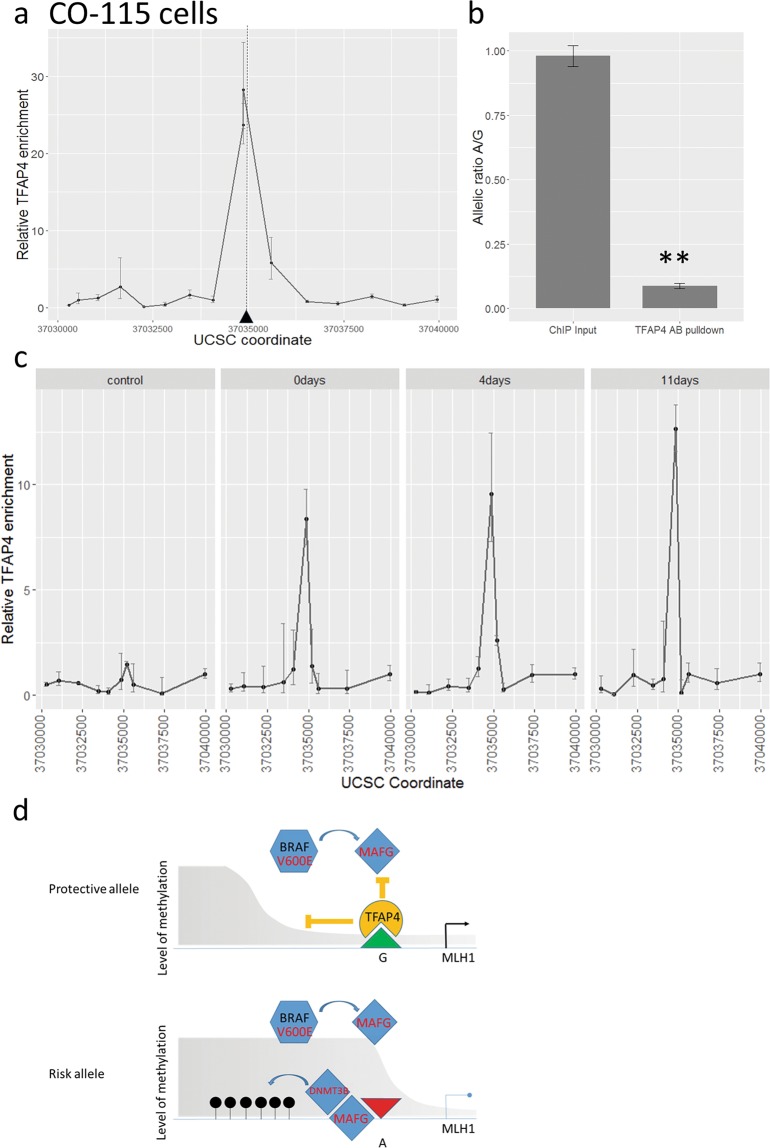
Allele specific binding of TFAP4 in the rs1800734 region in COLO320 (MSS) cells and induction in TFAP4 binding after AzaC treatment of CO-115 (MSI) cells. (**a**) Chromatin immunoprecipitation (ChIP) with TFAP4 in COLO320 cells shows an enrichment at rs1800734 (chr3:37034946 (hg19), black triangle). (**b**) Graph showing allele specific analysis of ChIP input and TFAP4 pull down with the input showing an unbiased A/G allelic ration and TFAP pull down showing a strong bias towards the G allele. Asterisks denote highly significant (p < 0.01) differences between alleles. (**c**) Linegraphs showing no TFAP4 binding in untreated CO-115 cells with enrichment seen at 4 days and 11 days post AzaC treatment. (**d**) Cartoon showing proposed mechanism by which rs1800734 influences MLH1 expression. (Upper Panel) The protective allele (G, green triangle, upper panel) binds TFAP4 (yellow) which protects the promoter (black arrow) from BRAF and MAFG (blue) directed methylation and/or methylation spreading from the CpG island shore. The grey shaded area represents methylation levels across the region. (Lower Panel). The risk allele (A, red inverted triangle) does not bind TFAP4 allowing MAFG and cofactors to mediate DNMT3B methylation in the promoter region causing transcriptional repression.

Liu *et al*.^13^ demonstrated in a cell-free system that the transcription factor TFAP4 binds *in vitro* to the protective (G) allele of rs1800734 with higher affinity than to the risk (A) allele. We hypothesized that preferential TFAP4 binding on the G allele may give some protective effect from *de novo* DNA methylation machinery. We therefore confirmed that in the heterozygous MSS cell line COLO320, TFAP4 binds strongly and in a localized fashion to the region containing rs1800734 (Fig. 4a). In addition, we showed a highly significant allele-specific bias in binding towards the G allele (Fig. 4b; p = 0.00295, t-test). We have also showed the TFAP4 binding location and allelic bias in a second heterozygous MSS cell line, CACO2 (Supplementary Fig. 5a,b; p = 0.0145, t-test). However, the MSI+ CO-115 cells in their methylated state had no detectable TFAP4 binding (Fig. 4c left panel) at rs1800734. Strikingly, after AzaC treatment to remove methylation, we observed strong TFAP4 binding in CO-115 at the rs1800734 in all post-treatment time-points (Fig. 4c middle and right panels), reflecting the methylation and *MLH1* expression changes seen in Fig. 3. We additionally discounted any significance of rs1800734 genotype on the expression of *DCLK3* 280 kb upstream of *MLH1*, contrary to previous reports^13^ (Supplementary Fig. 6).

## Discussion

We have confirmed that the SNP rs1800734 in the promoter of *MLH1* is associated with the risk of sporadic MSI+ CRC, but has no effect on MSS risk. This strong influence on the mismatch repair pathway and the repressive effect of the rs1800734 risk allele on transcription of the mismatch repair pathway protein, MLH1, confirms this as the target gene. Our results demonstrate that the risk (A) rs1800734 allele has no measurable repressive effect on MLH1 in normal bowel tissues, even using highly sensitive techniques, and in fact associates with reduced DNA methylation at the upstream CpG island shore, in line with previous observations^12^. However, significant allele specific effects are seen on both methylation and mRNA expression in MSI+ cancers in our own and TCGA data sets. As expected, the risk (A) allele leads to significantly higher methylation levels and this strongly correlates with lower mRNA expression. The data of Fennell *et al*.^18^ and our small set of SSAs indicate that the allele methylation and expression bias probably arise very close together, during the serrated pathway.

We have also clearly demonstrated that DNA methylation is necessary for *MLH1* transcriptional silencing by treating with AzaC, removing methylation and demonstrating re-expression of *MLH1* in MSI+ cells. This result implies that methylation is indeed the primary cause of *MLH1* silencing in sporadic MSI+ cancers. Interestingly, even in this engineered situation the risk (A) allele is more prone to acquire methylation and its mRNA is re-expressed at lower levels. Thus it is likely that the mechanism by which rs1800734 mediates cancer risk is via methylation acquisition. The reason the A allele is more readily methylated could be due to the lack of binding of the TFAP4 transcription factor as shown by Liu *et al*.^13^. We confirm their result that there is indeed a strong bias in TFAP4 binding in unmethylated CRC cells and that this binding is dependent on promoter demethylation post AzaC treatment in MSI+ cells.

It is unlikely that TFAP4 is the only transcription factor binding across rs1800734 and publically available genome wide ChIP-seq experiments show multiple proteins binding in the region (ENCODE, UCSC). Indeed, TFAP4 has been shown to belong to a class of enhancer binding factors that are important for co-factor recruitment and activation^20^. This suggests that it could be a major factor in determining protein binding and potentially chromatin landscape across the region. The fact that TFAP4 also binds allele specifically to another disease associated SNP (rs12722522, Type 1 diabetes^21^) suggests it might play a more generalised role in a subset of SNP trait associations, acting to recruit activating factors in an allele specific manner. Thus, as our data suggest, the effect of TFAP4 on *de novo* methylation of the MLH1 promoter is unlikely to be direct and therefore may only offer partial protection such that the rs1800734 protective (G) allele can still acquire methylation but at a slower rate or at lower levels than the risk (A) allele.

Liu *et al*.^13^ suggest that allele specific TFAP4 binding at rs1800734 may exert an effect on the cancer pathway via long range interactions with the promoter of the DCLK3 gene, causing enhanced expression of genes related to epithelial-to-mesenchymal transition. However we were unable to corroborate this finding. We failed to detect significant DCLK3 expression in either our MSS or MSI+ cell lines using sensitive Q-PCR based techniques. We have clearly demonstrated that rs1800734 is only associated with an increased risk of MSI+ cancers, ie those with a dysfunctional MMR pathway, and only modifies methylation in these and not in MSS cancers. Thus, it seems unlikely that a gene with no known role in MMR plays the primary or causative role in rs1800734 associated cancer risk.

Our data, taken together with other studies described above, support the prevailing hypothesis that MLH1 repression is the main mechanism by which rs1800734 confers cancer risk. Since the majority of our MSI+ cancers (23/35) and all our SSA samples also carry a BRAFV600E mutation we suggest it is likely that rs1800734 influences the acquisition of methylation via the BRAF/MAFG pathway described by Feng *et al*.^4^. Figure 4d represents our proposed model in which TFAP4, with co-factors binds within the promoter region on the G protective allele. This restricts the access of the BRAF activated MAFG complex and consequently reduces the spread of DNA methylation from the CpG island shore. On the A allele TFAP4 binding occurs less frequently or with lower affinity allowing MAFG access and methylation spreading.

While the functional role of rs1800734 in the pathways of CRC development is becoming clearer, it is also interesting to note how readily the accumulation of MLH1 promoter methylation could be reversed resulting in re-expression of MLH1. The importance of MLH1 promoter methylation in other cancer types is less well understood however it is frequently found in endometrial^22^, and gastric^23^) cancers, as well as lung^24^, bladder^25^, and some haematological malignancies^26^. Drugs such as Azacitidine that inhibit DNA methylation are already approved for the treatment of some cancers. However with the advent of CRISPR technology, more precise demethylation is now possible^27^ and could be harnessed in the design of future therapies.

## Methods

### Patient samples

A summary of all patient sample sets used in this study is given in Supplementary Fig. 7. Patient samples for genetic studies were as reported in Allan *et al*., Campbell *et al*., Raptis *et al*., Whiffin *et al*.^5–8,28^ plus our own dataset using clinical trial samples (QUASAR2^29^ and VICTOR^30,31^). All patients were genotyped on Illumina tagSNP or custom arrays and quality control was performed as previously described.

Normal colorectal biopsies from 317 individuals of white UK origin undergoing colonoscopy in Oxford as previously reported^32^, were used for allele specific analysis of methylation and MLH1 mRNA expression. Formalin Fixed Paraffin Embedded (FFPE) CRC MSI+ tumours for analysis of methylation, expression and rs1800734 were collected at the Department of Pathology of ASST dei Sette Laghi- University of Insubria. 35 consecutive sporadic CRCs showing MSI and MLH1/PMS2 immunohistochemical loss were selected^33^. All CRCs were histologically reviewed according to the World Health Organization (WHO) classification of tumors of the digestive system^34^ and the TNM staging system^35^ These patients were 20 females and 15 males with a mean age at CRC diagnosis of 77 years (range: 55–88 years). Thirty-two neoplasms arose in right colon, one in descending colon and the remaining two cases in sigmoid colon. Sessile serrated adenomas were collected fresh and frozen following endoscopic resection.

### Ethical review

Collection of blood and tissue samples and clinico-pathological information from patients and controls was undertaken with informed consent and ethical review board approval in accordance with the tenets of the Declaration of Helsinki.

Victor and Ethical approval for the QUASAR study was obtained from the West Midlands Research Ethics Committee (Edgbaston, Birmingham, UK; REC reference: 04/MRE/11/18). The VICTOR trial was reviewed by the Cancer Research Campaign, the Multicenter Research Ethics Committee, and research ethics committees at participating centers. Ethical approval for the normal patient samples was obtained from the Oxfordshire Research Ethics Committee A (REC 10/H0604/72). The formalin fixed paraffin embedded (FFPE) CRC MSI+ tumours sample study was approved by the Ethics Committee of Ospedale di Circolo di Varese (No. 0037028). The sessile serrated adenomas was approved by Oxford Radcliffe Biobank ethics committee (09/H0606/5 + 5 ORB biobank, 13/SL/Precursor-lesions).

### SNP genotyping

Patient samples and cell lines were genotyped for rs1800734 using KASP^TM^ technology according to the manufacturer’s instructions (LGC). Primers are listed in Supplementary Table 4.

### Analysis of methylation

DNA was extracted from fresh cells or tissue using the DNeasy kit (QIAGEN) or from FFPE tissue using the High Pure FFPET DNA Isolation Kit (Roche). Bisulphite conversion of DNA was carried out using the EZ DNA methylation kit (Zymo Research) according to the manufacturer’s instructions. Converted DNA was amplified with Pyromark PCR kit (Qiagen) using CpG free primers with Illumina specific sequence tags to ensure unbiased amplification of methylated and unmethylated template (Supplementary Table 4). Amplicons from each patient were barcoded together using a custom set of index tags and primers^36^. Up to 96 samples were sequenced using 250 bp paired end sequencing on a MiSeq (Illumina) according to the manufacturer’s instructions. MiSeq output was demultiplexed and FASTQ files generated (Basespace, Illumina). The sequences were quality assessed, and trimmed (FastQC and TrimGalore, Babraham Bioinformatics) then aligned and the methylation called (Bismark, Babraham Bioinformatics).

MLH1 Methylation analysis on FFPE CRCs was performed in two replicates for each sample by MS-MLPA using the SALSA MS-MLPA ME011-Mismatch Repair genes kit (version B3) (MRC-Holland, Amsterdam, The Netherlands). MS-MLPA was performed according to the manufacturer’s instructions and data analysis was carried out with Coffalyser software v.8 (MRC-Holland).

### Analysis of mRNA

RNA was extracted from fresh cells or tissue using the RNeasy kit (QIAGEN) or from FFPE tissue using the High Pure FFPET RNA Isolation Kit (Roche) and cDNA was generated (High Capacity cDNA Reverse TRAnscription Kit, Applied Biosystems) according to the manufacturer’s instructions. Gene expression was quantified and normalized using Taqman gene expression ready mixed assays (Applied Biosystems, Thermofisher). Allele specific MLH1 expression was assessed by amplification of cDNA using Illumina tagged primers (Supplementary Table 4) followed by NGS sequencing on a MiSeq (Illumina) as above. Trimmed FastQ sequences were aligned using bwa-mem and the rs1800734 variant called by Platypus^37^.

### Cell culture

Cell lines were grown in Dulbecco Modified Eagles Medium or RPMI-1640 supplemented with 10% fetal bovine serum and 1% penicillin streptyomycin (Sigma) at 37 °C in 5% CO_2_.

### Chromatin Immunoprecipitation

Approximately 10^8^ cells were crosslinked for 10 mins with 1% formaldehyde, neutralized with 125 mM glycine, washed with ice-cold PBS and scraped. After 2 further PBS washes, cells were resuspended in lysis buffer, (1% SDS, 10 mM EDTA, 50 mM Tris-HCl, protease inhibitors) sonicated using a Bioruptor (Diagenode) for 7–15 × 15 s cycles, centrifuged at max speed for 10 min at 4 °C and diluted 1:10 in IP dilution buffer (1% triton-100, 2 mM EDTA, 150 mM NaCl, 20 mM Tris). Immunoprecipitation with approximately 5 ug of antibody (anti-TFAP4 Santa Cruz Biotechnology, sc-18593x) was carried out overnight at 4 °C and then incubated for 4 hours with 50 ul of protein G Dynabeads (Invitrogen). Bead/antibody complexes were washed with TSEI (0.1% SDS, 1% TritonX-100, 2 mM EDTA, 20 mM Tris, 150 mM NaCl), TSEII (0.1% SDS, 1% TritonX-100, 2 mM EDTA, 20 mM Tris, 500 mM NaCl), LiCl buffer (0.25LiCl, 1% NP-40, 1% deoxycholate, 1 mM EDTA, 10 mM Tris-HCl) and TE according to standard protocols and eluted with 1% SDS, 0.1 M NaHCO_3_. 1 ul of DNA was analyzed in duplicate or triplicate by SYBR green qPCR using PowerUp SYBR™ Green Master Mix (Thermofisher) and primers covering the MLH1 promoter region (Supplementary Table 5).

### 5-Aza-2′-deoxycytidine treatment

Adherent semiconfluent MSI+ cells in exponential growth were treated with 5 uM 5-Aza-2′-deoxycytidine in standard medium (AzaC, Sigma A3656) for 48 hours (with replenishment of AzaC after 24 hours). AzaC was removed, cells washed with PBS, and then cultured in standard medium for 0, 4, 7 and 11 days. RNA and DNA were extracted simultaneously using the AllPrep kit (Qiagen) and MLH1 mRNA expression and promoter methylation assessed as described above. ChIP was carried out post AzaC treatment as described above.

### Statistical analysis

SNP association studies were carried out using PLINK. All other statistical analysis was carried out using R unless otherwise stated. Graphs were drawn using core R functions, ggplot2 or excel.

## Supplementary information

Supplementary Information

Supplementary tables

## Data Availability

The raw data is available on Mendeley at https://data.mendeley.com/datasets/hfpbctm7tg/draft?a=1c91e494-cadc-4be0-a8ff-91d8736a28e7.
