# Supplementary material for: The polymorphic variant rs1800734 influences methylation acquisition and allele-specific TFAP4 binding in the MLH1 promoter leading to differential mRNA expression
Source: Sci Rep. 2019 Sep 17;9:13463. doi: 10.1038/s41598-019-49952-x (PMC6748923; doi:10.1038/s41598-019-49952-x)
Supplement: Supplementary file 1 — Supplementary Information [file 41598_2019_49952_MOESM1_ESM.docx]

**Supplementary information for:**

**The polymorphic variant rs1800734 influences methylation acquisition and allele-specific TFAP4 binding in the MLH1 promoter leading to differential mRNA expression.**

Rachael Thomas*^1^, Davide Trapani*^2^, Lily Goodyer-Sait^3^, Marketa Tomkova^4^, Ceres Fernandez-Rozadilla^5^, Nora Sahnane^2^, Connor Woolley^6^, Hayley Davis^7^, Laura Chegwidden^8^, Skirmantas Kriaucionis^4^, Timothy Maughan^9^, Simon Leedham^7^, Claire Palles^8^, Daniela Furlan^2^, Ian Tomlinson^6^, Annabelle Lewis^1#^

**Supplementary Information**


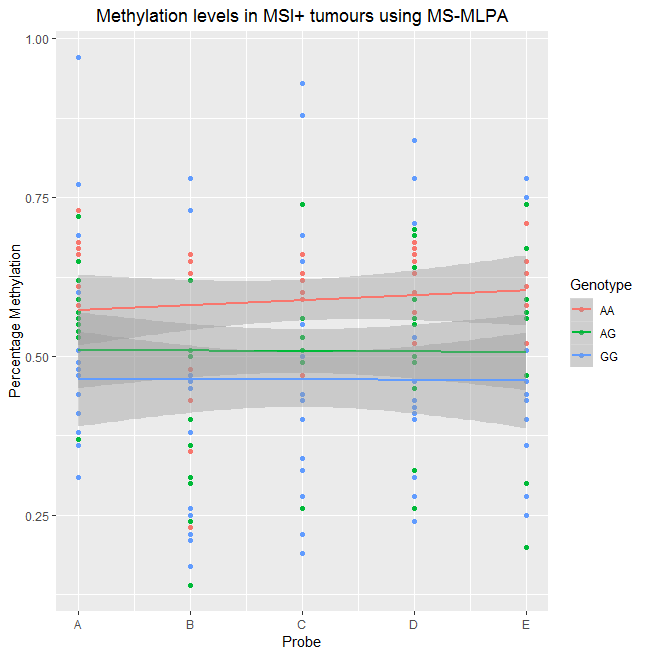
Supplementary Figure 1: **Methylation levels in MSI+ tumours using MS-MLPA**. 5 probes were used reporting each Deng methylation region ((Deng et al., 1999) Scatter plot grouped by genotype with Loess curves and SE shaded with grey (n= 35, Supplementary table 3)

**
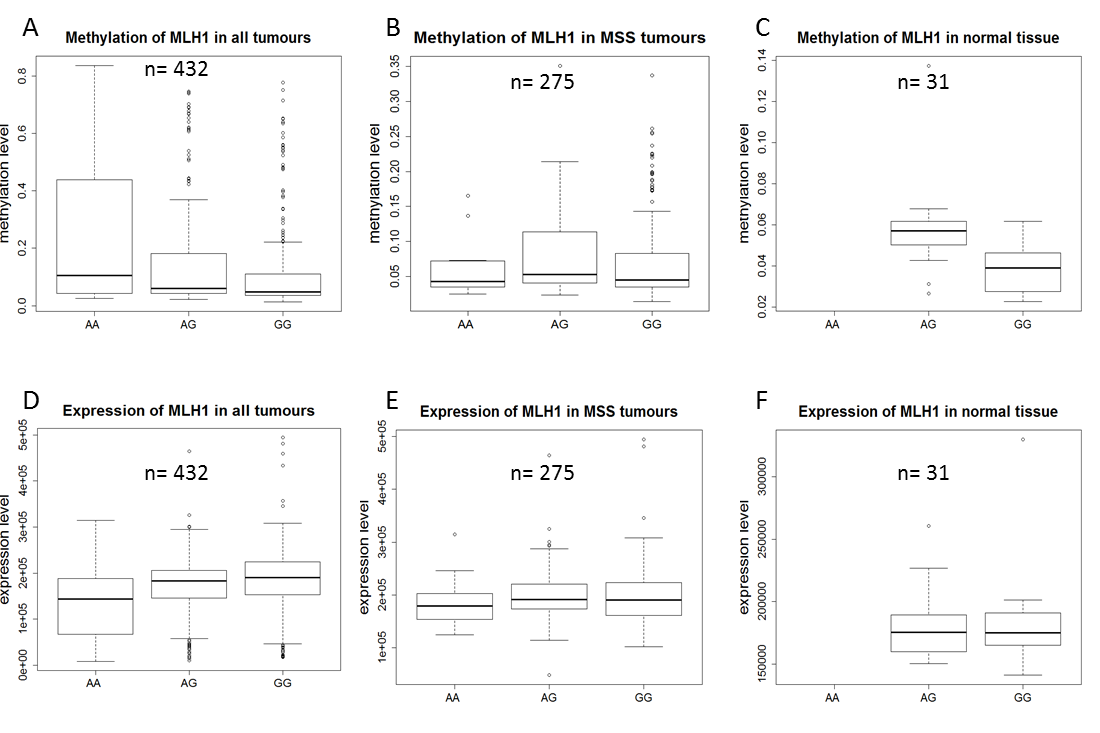
**Supplementary Figure 2: **Methylation (A-C) and expression (D-F) of MLH1 in TCGA COADREAD samples stratified by rs1800734 genotype.**

Supplementary Figure 3: **Methylation and expression levels in sessile serrated adenomas (SSAs)**: (A) scatter plot showing allele specific methylation levels across CpGs close to rs1800734 with samples grouped by allele. Loess curves for each allele are shown with standard error shaded in grey, rs1800734 is marked by a black triangle (n=4); (B) boxplot showing *MLH1* allelic mRNA expression ratio (A/G) in heterozygous samples comparing normal cDNA (n=41) with SSA cDNA (n=5). Methylation patterns in SSAs are distinct from MSI+ cancers and normal samples with an increase in methylation biased towards the A allele close to the CpG island shore but not yet around rs1800734. Expression patterns show a significant bias towards the G allele.


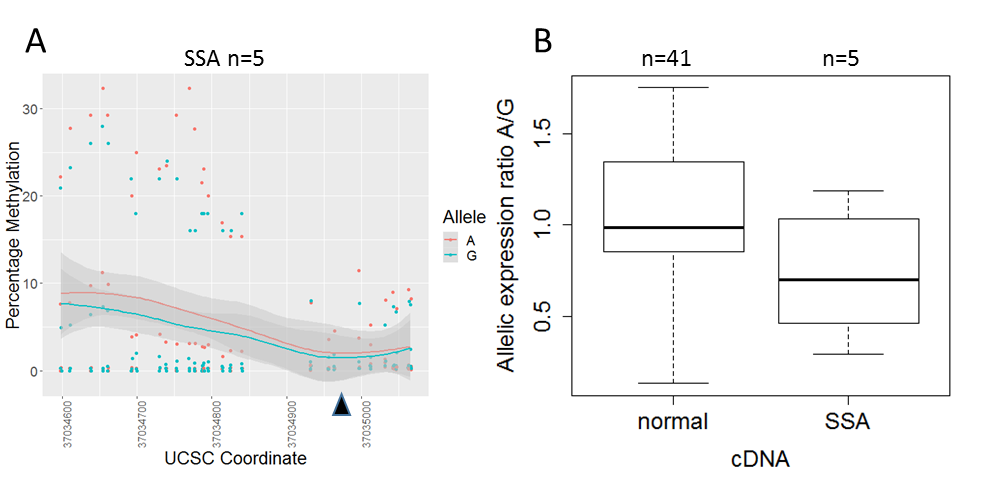


Supplementary figure 4: **Increase in MLH1 expression levels in SW48 (MSI+, MLH1 promoter hypermethylation, BRAF wildtype) cells after treatment with AzaC.** Barchart showing total MLH1 mRNA expression in control untreated cells and timepoints post AzaC treatment.


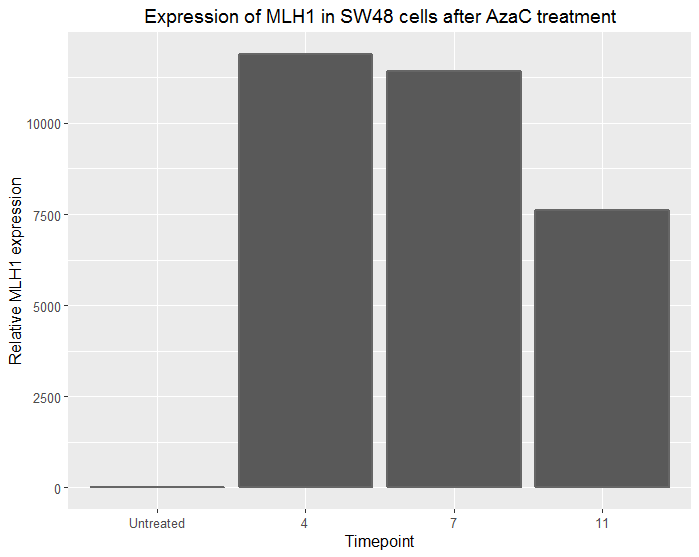


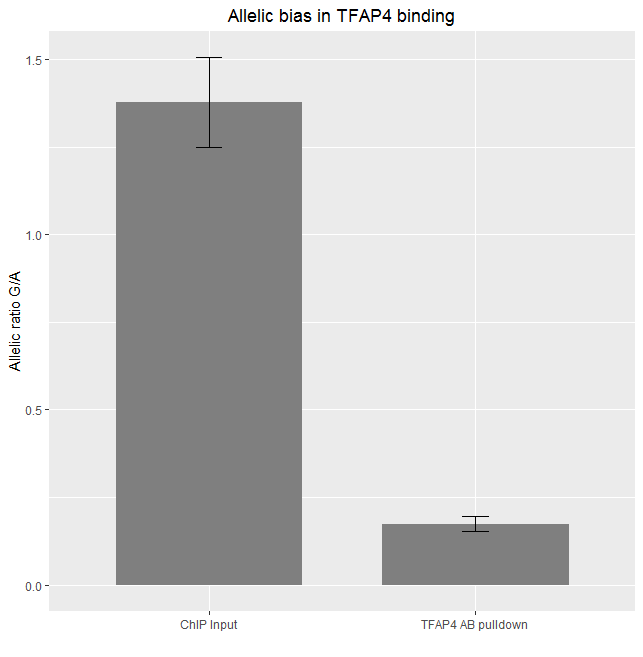

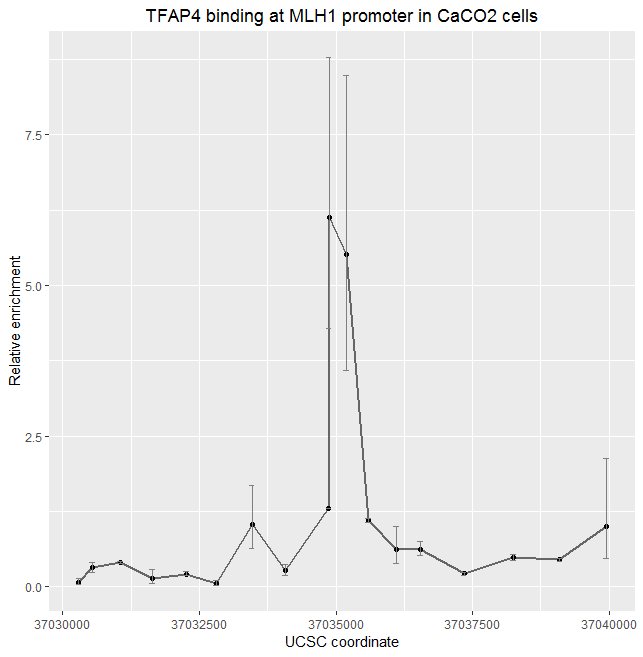
Supplementary figure 5: **TFAP binds to rs1800734 in CACO2 cells (MSS, MLH promoter unmethylated, BRAF wildtype) with a strong bias towards the protective (G) allele.**

B

A

**


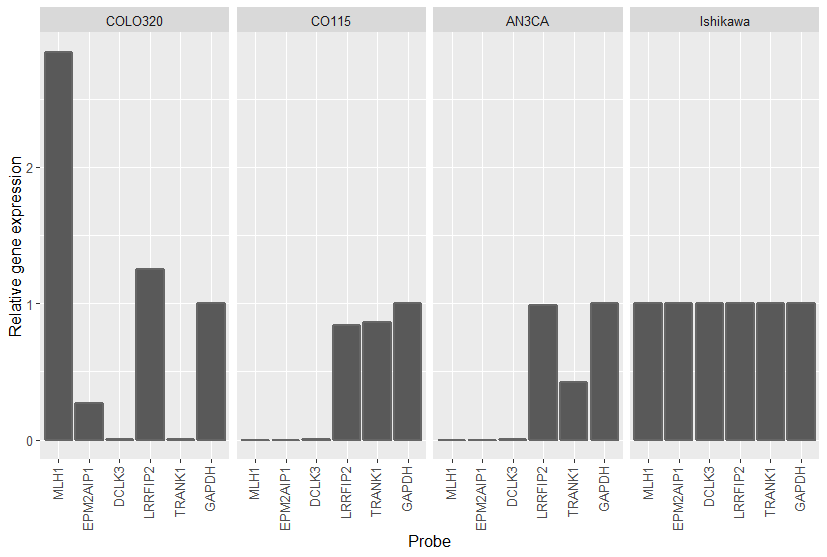
Supplementary figure 6: **Dclk3 expression is undetectable in COLO320 and other CRC cell lines**. cDNA from cell lines was amplified using Taqman (Applied Biosystems) probes. Expression levels were normalized to a GAPDH control probe and expressed relative to levels in the EC Ishikawa cell line using the ΔΔCt method. MLH1 and EPM2AP1 are expressed in the MSS COLO320 cell lines but not in MSI+ CO115. DCLK3 is undetectable in these and other CRC cell lines although other genes in the region show expression. DCLK3 expression is detected in Ishikawa cells as a positive control.

Supplementary figure 7: **Flow chart describing samples sets used in this study**


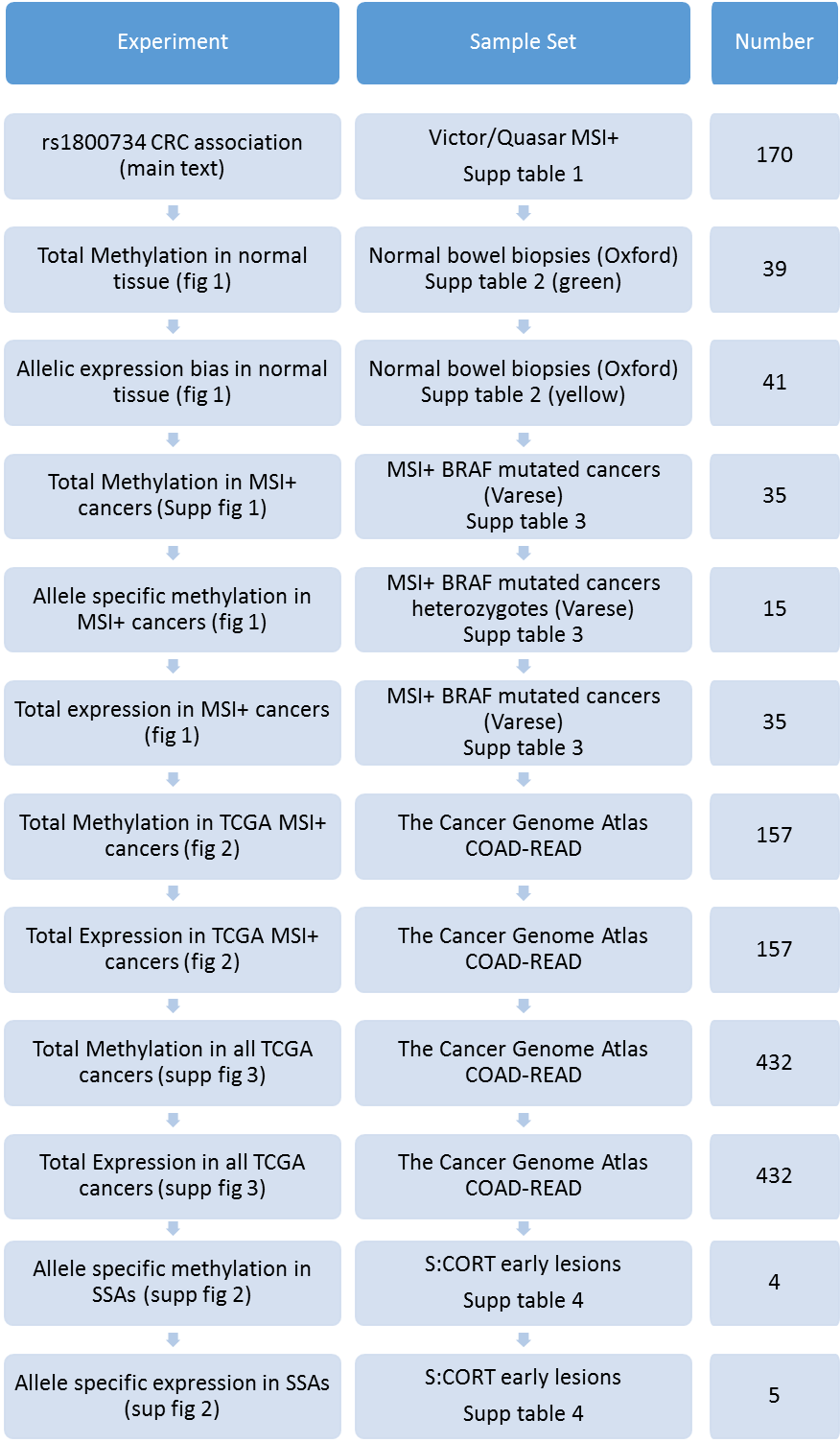


Supplementary table 5: **Primers used for genotyping, amplicon bisulphite sequencing and cDNA amplification**

Supplementary table 6: **Primers used for Q-PCR (SYBR) amplification of ChIP DNA**

Deng, G., Chen, A., Hong, J., Chae, H.S., and Kim, Y.S. (1999). Methylation of CpG in a small region of the hMLH1 promoter invariably correlates with the absence of gene expression. Cancer Res *59*, 2029-2033.
